# Pleural inflammatory myofibroblastoma: a locally aggressive intra-thoracic tumour

**DOI:** 10.1186/1749-8090-2-29

**Published:** 2007-06-28

**Authors:** Chandrashekhar Kubal, Sanjay Ghotkar, John Gosney, Martyn Carr

**Affiliations:** 1 Department of Cardiothoracic Surgery, Cardiothoracic Centre, Thomas Drive, Liverpool, L14 3PE, UK; 2 Department of Renal Transplantation, University Hospital Birmingham, Edgbaston, Birmingham, B15 2TH, UK; 3 Department of Pathology, 5th floor, Duncan Building, Royal Liverpool University Hospital, Daulby Street, Liverpool, L69 3GA, UK

## Abstract

A 41-year old non-smoking woman presented with persistent pleural effusion. Pleural fluid was hemorrhagic and fluid cytology was negative for malignant cells. A working diagnosis of chronic haemothorax was made and standard right thoracotomy was performed to identify the source of bleeding. A 10 × 10 cms poorly circumscribed mass containing blood clots, altered blood, fibrous tissue, and gelatinous debris was found and demonstrated features of inflammatory myofibroblastoma on immunohistochemistry. Thirteen months later, the patient developed a local recurrence, which was treated surgically. Semi-solid physical appearance of this tumour has not been reported previously. This case report further adds to the diagnostic dilemma related with this tumour.

## Background

Inflammatory myofibroblastic tumour is a rare entity. These tumours are well known for their bizarre presentation and behaviour. This case report further supports their variability in presentation. We present a case of pleural inflammatory myofibroblastoma that presented as a chronic haemothorax.

## Case presentation

**Figure 1:**
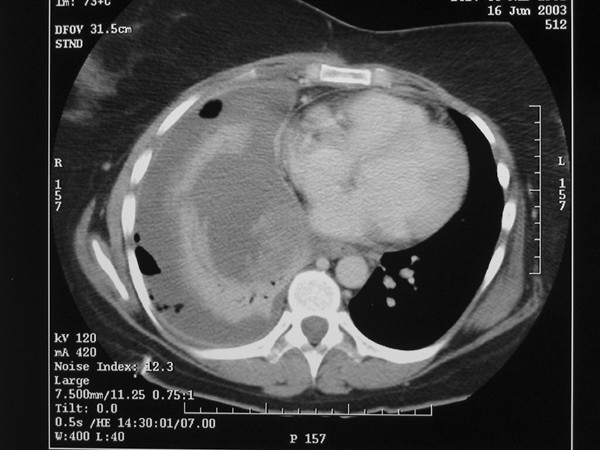
C T scan showing right-sided pleural effusion along with compressive atelectasis in the right lower lobe without thickening of visceral or parietal pleura.

A 41-year-old non-smoking woman presented at a district general hospital with generalised weakness, breathlessness and non-productive cough for 5–7 weeks. Chest x-ray revealed a moderate right-sided pleural effusion, which was drained with a pigtail catheter. The drained fluid was hemorrhagic and fluid cytology was negative for malignant cells. The patient then became generally unwell, pyrexial and anaemic with increasing effusion on serial chest films. A C.T. scan of the chest [Figure 1] showed right-sided pleural effusion along with compressive atelectasis in the right lower lobe without thickening of visceral or parietal pleura. Serial pleural fluid cultures were negative. Due to continuing diagnostic uncertainty and falling haemoglobin levels, the patient was referred to our thoracic surgery unit. On admission to our unit laboratory analysis revealed haemoglobin 7.0 g/dl, WBC 7000/mm3, and ESR 15 mm/hr. A diagnosis of chronic haemothorax was made and standard right thoracotomy was performed. After mobilising the right lower lobe from diaphragm a vaguely defined mass was identified lying between the diaphragmatic surface of the right lower lobe and right hemi diaphragm. This mass was approximately 10 × 10 cms in size, poorly circumscribed and contained blood clots, altered blood, fibrous tissue, and gelatinous debris. After removal of the mass the right lower lobe expanded well, and no other lesions were found in the right lung and parietal pleura. The patient recovered well from the operation with no recurrence of pleural effusion.

However, 13 months later, the patient presented again with an increasing right-sided pleural effusion. A CT scan revealed a soft tissue mass in right costophrenic recess. This was thought to be due to recurrence of the initial tumour and right thoracotomy was performed. A poorly circumscribed gelatinous tumour measuring 5 × 7 cms was present in the right costophrenic angle involving adjacent diaphragm, pericardium and visceral pleura of the right lower lobe. A right lower lobectomy was performed along with resection of right hemi diaphragm and involved pericardium. The defects were repaired with a patch. One year later, the patient was asymptomatic and CT-scan revealed no tumour recurrence.

## Histopathological findings

**Figure 2:**
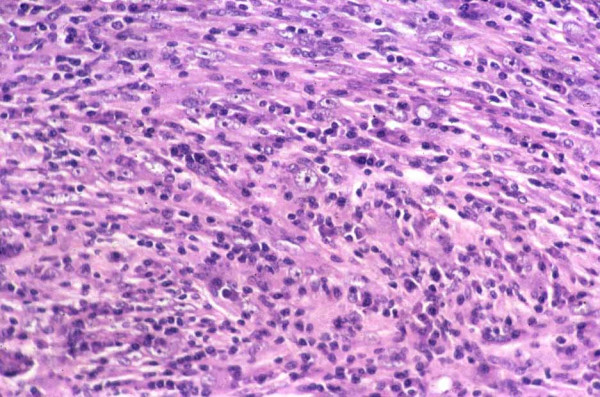
Hematoxylin and eosin staining demonstrating neoplastic proliferation of fusiform cells with pleomorphism and occasional tumour 'giant cells'. These features were typical of inflammatory myofibroblastoma.

**Figure 3:**
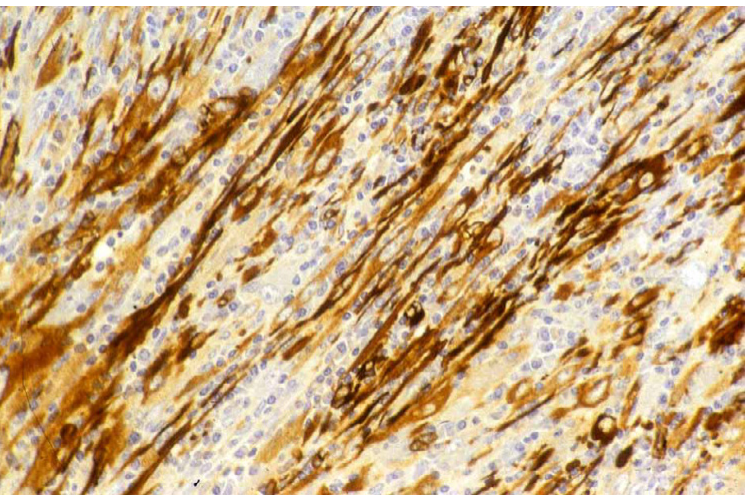
Immunohistochemistry demonstrating strong expression of vimentin and smooth muscle actin.

Histological examination of the initial and recurrent tumour revealed a neoplastic proliferation of fusiform cells with pleomorphism and occasional tumour 'giant cells' [Figure 2]. The stroma was myxoid with an inflammatory infiltrate. Immunohistochemistry revealed strong expression of vimentin and smooth muscle actin [Figure 3]. There was no expression of cytokeratins. These features were typical of inflammatory myofibroblastoma. The specimen was received piece meal due to semi-solid consistency of the tumour. Therefore it was not possible to assess resection margins for tumour involvement. The lymph nodes resected demonstrated reactive changes only.

## Discussion

Inflammatory myofibroblastoma can occur in many parts of the body. Its histological elements are myofibroblasts, histiocytes, plasma cells, and lymphocytes. Presence of these features suggests that this is a fibro-inflammatory lesion with abnormal extension of the reparative healing process after an unknown insult. Inflammatory myofibroblastoma is rarely found in the pleural space. It is also uncommon amongst tumours of the lung with an incidence varying from 0.04% [1] to 0.7% [2], but is rare in the pleural space. It has also been reported to arise from diaphragm [3], heart, and many extra-thoracic locations such as liver, spleen, testes, epididymis, spermatic cord, bladder, salivary glands, spinal meninges and orbit.

The characteristics of this tumour include its bizarre presentation and unknown natural history, as in the case presented. This tumour may have variable physical consistency. Apart from semi-solid gelatinous consistency presented in this case it can also present as a solitary nodule [4] and solitary or multiple calcified fibrous nodules [5]. It can easily be confused with lung cancer when it appears as a solitary nodule on CT scan. Both benign and malignant pleural conditions can be confused with the non-solid presentation of this tumour. The case of inflammatory myofibroblastoma presented in this case report is unique due to its presentation as a long-standing haemothorax and it's non-solid physical appearance. This only adds to the diagnostic dilemma related with this tumour. Surgical treatment can be challenging in non-solid tumours, as complete removal can be difficult; resulting in a high risk of local recurrence. Distant recurrence has been reported as early as 3 months in the form of multiple bilateral pulmonary nodules and occurrence in multiple organs after lung resection has been reported [6]. On the other hand two different series consisting of seven patients each have reported no evidence of recurrence with follow-up ranging from 0.5–13 yrs [7,8].

This case report emphasizes the difficulty in making preoperative diagnosis of pleural inflammatory myofibroblastoma. This lesion should be kept in mind when dealing with non-resolving haemorrhagic pleural effusions in absence of a suspicious malignant lesion. A low threshold to perform CT guided biopsy may offer preoperative diagnosis and better planning of surgical management in these tumours.

## Competing interests

The author(s) declare that they have no competing interests.

## Authors' contributions

CK and SG carried out the data and information collection along with designing and writing the manuscript draft; JG and MC provided help with designing and writing the manuscript. All authors read and approved the final manuscript.
